# Soluble CD14 Enhances the Response of Periodontal Ligament Stem Cells to *P*. *gingivalis* Lipopolysaccharide

**DOI:** 10.1371/journal.pone.0160848

**Published:** 2016-08-09

**Authors:** Oleh Andrukhov, Olena Andrukhova, Burcu Özdemir, Hady Haririan, Michael Müller-Kern, Andreas Moritz, Xiaohui Rausch-Fan

**Affiliations:** 1 Division of Conservative Dentistry and Periodontology, University Clinic of Dentistry, Medical University of Vienna, Vienna, Austria; 2 Department of Biomedical Science, University of Veterinary Medicine, Vienna, Austria; 3 Department of Periodontology, Faculty of Dentistry, Gazi University, Ankara, Turkey; Katholieke Universiteit Leuven Rega Institute for Medical Research, BELGIUM

## Abstract

Periodontal ligament stem cells (PDLSCs) are lacking membrane CD14, which is an important component of lipopolysaccharide (LPS) signaling through toll-like receptor (TLR) 4. In the present study we investigated the effect of soluble CD14 on the response of human PDLSCs to LPS of *Porphyromonas (P*.*) gingivalis*. Human PDLSCs (hPDLSCs) were stimulated with *P*. *gingivalis* LPS in the presence or in the absence of soluble CD14 (sCD14) and the production of interleukin (IL)-6, chemokine C-X-C motif ligand 8 (CXCL8), and chemokine C-C motif ligand 2 (CCL2) was measured. The response to *P*. *gingivalis* LPS was compared with that to TLR4 agonist *Escherichia coli* LPS and TLR2-agonist Pam3CSK4. The response of hPDLSCs to both *P*. *gingivalis* LPS and *E*. *coli* LPS was significantly enhanced by sCD14. In the absence of sCD14, no significant difference in the hPDLSCs response to two kinds of LPS was observed. These responses were significantly lower compared to that to Pam3CSK4. In the presence of sCD14, the response of hPdLSCs to *P*. *gingivalis* LPS was markedly higher than that to *E*. *coli* LPS and comparable with that to Pam3CSK4. The response of hPdLSCs to bacterial LPS is strongly augmented by sCD14. Local levels of sCD14 could be an important factor for modulation of the host response against periodontal pathogens.

## Introduction

Mesenchymal stem cells (MSCs) are multipotent cells that are able to differentiate in osteoblasts, adipocytes, and chondrocytes and express characteristic pattern of surface markers [1]. MSC-like cells are found in all dental tissues, including dental pulp, gingival tissue, periodontal ligament and others [2,3]. Periodontal ligament stem cells (PDLSCs) have a fibroblast-like shape, express characteristic mesenchymal stem cells surface markers, and exhibit the ability to differentiate into different cell types *in vitro* [4]. As recently reviewed, PDLSCs play an important role in periodontal tissue homeostasis and regeneration and similar to MSCs also possess immunomodulatory ability [5]. Noteworthy, cells isolated from periodontal ligament are used in research in numerous studies (for review, see [6]). The common characteristic of these cells is their ability to differentiate into osteoblasts [7] and therefore might contain some population of multipotent progenitor cells. It is rather difficult to discriminate mesenchymal stem cells from common fibroblasts *in vitro*, because both cell types share common surface markers [8].

Periodontitis is an inflammatory disease leading to periodontal tissue destruction and eventually to tooth loss [9,10]. Periodontitis is associated with gram-negative bacteria in the dental pocket but the major reason of tissue destruction seems to be an inappropriate unregulated host response to bacterial pathogens [11]. *P*. *gingivalis* is a gram-negative bacteria and plays an important role in the development of periodontitis [12]. *P*. *gingivalis* LPS is considered as one of the major virulence factor of these bacteria. The structure and virulence activity of *P*. *gingivalis* LPS is strikingly different from those of most gram negative bacteria, it contains tetra- and pentaacetylated lipid A species and might activate both toll like receptor (TLR) 2 and TLR 4 [13,14]. In contrast, LPS of common enterobacteria *E*. *coli* activates only TLR4 [15]. The activation of TLR2 by *P*. *gingivalis* LPS is sometimes related to insufficient purity of LPS preparations (e.g. [16]).

As recently reviewed, mesenchymal stem cells might be involved in the progression of periodontal disease and their immunomodulatory properties are considered as a potential therapeutic approach for periodontitis [17]. Therefore, interaction of periodontal pathogens, particularly *P*. *gingivalis* LPS, with periodontal ligament stem cells might play an important role in the progression of periodontal disease but it was not investigated intensively. A recent study shows that prolonged treatment of hPDLSCs with *P*. *gingivalis* LPS enhances the production of IL-6 and CXCL8 by human PDLSCs and inhibits their osteogenic differentiation [18]. The inhibition of osteogenic differentiation by LPS seems to be mediated through TLR-4 and might be associated with an increased expression of miRNA-138 [19]. Interestingly, the most pronounced response of PDLSCs to LPS is observed at concentration of 10 μg/ml, whereas at concentrations up to 1 μg/ml LPS is not always effective [18]. The low reactivity of PDLSCs to bacterial LPS could be explained by the fact that these cells by definition do not express membrane bound CD14 [5], which is an important co-factor of TLR-4 activation by LPS [20]. Beside membrane form, there is also a soluble form of CD14 [21]. We hypothesized that soluble CD14 might influence the response of human PDLSCs to *P*. *gingivalis* LPS. In the present study, we investigated the effect of soluble CD14 on the production of IL-6, CXCL8, and CCL2 by human PDLSCs in response to *P*. *gingivalis* LPS. The effect of *P*. *gingivalis* LPS was compared with that of TLR-4 agonist *E*. *coli* LPS and TLR-2 agonist Pam3CSK4.

## Material and Methods

### Cell Culture and Reagents

Primary periodontal ligament cells were isolated from periodontally healthy patients aged 18–20 years undergoing routine extraction of their third molar teeth. Patients were informed in detail before the surgical procedures and gave their written agreement. The study protocol was approved by the Ethics Committee of the Medical University of Vienna. Periodontal ligament tissue was scraped from the teeth root surface with a scalpel, cut into small pieces and digested by collagenase/dispase (Sigma, St. Louis, MO, USA) for 30 min at 37°C. Cells were cultured in Dulbecco’s modified Eagle’s medium (DMEM), supplemented with 10% fetal bovine serum (FBS), streptomycin (50 μg/ml) and penicillin (100 U/ml) under humidified air atmosphere of 5% CO_2_ at 37°C. Cells isolated from 7 different donors were used; cells from passage levels 3–6 were used in the experiments.

Commercially available *P*. *gingivalis* LPS (standard preparation), *E*. *coli* LPS (ultrapure preparation), and TLR-2 agonist Pam3CSK4 were purchased from Invivogen (San Diego, USA). Human soluble CD14 was purchased from Sigma (St. Louis, MO, USA).

### Characterization of periodontal ligament stem cells by flow cytometry

hPDLSCs were stained with one of the following monoclonal antibodies (all from eBiosciences, San Diego, CA, USA): phycoerythrin (PE)-conjugated mouse anti-human CD29, PE-conjugated mouse anti-human CD90, PE-conjugated mouse anti-human CD105, PE-conjugated mouse anti-human CD146, PE-conjugated mouse IgG1 K isotype control, fluorescein isothiocyanate (FITC)-conjugated mouse anti-human CD14, FITC-conjugated mouse anti-human CD31, FITC-conjugated mouse anti-human CD34, FITC-conjugated mouse anti-human CD45, FITC-conjugated mouse IgG1 K isotype control.

### Stimulation protocol

Primary hPDLSCs were seeded in a 24-well plate at a density of 5x10^4^ cells per well containing 0.5 mL of DMEM medium supplemented with 10% FBS and 1% P/S. After 24 h, medium was changed to serum-free DMEM with 1% P/S. Cells were stimulated with *P*. *gingivalis* LPS (0.01–1 μg/ml), *E*. *coli* LPS (1 μg/ml), or Pam3CSK4 (1 μg/ml) in the presence or in the absence of sCD14 (250 ng/ml) for 24 h. In some experiments, sCD14 was used also in concentrations of 2.5 and 25 ng/ml. After stimulation, the cellular mRNA expression levels of IL-6, CXCL8, and CCL2 in cells as well as the content of corresponding proteins in the conditioned media were determined.

### Quantitative PCR and ELISA

The mRNA expression levels of IL-6, CXCL8, and CCL2 were determined by qPCR as described previously [22,23], taking the β-actin encoding gene as internal reference. Isolation of mRNA and transcription into cDNA was performed using the TaqMan Gene Expression Cells-to-CT kit (Ambion/Applied Biosystems, Foster City, CA, USA) according to the manufacturer’s instructions. This kit provides good accuracy and superior sensitivity of gene-expression analysis [24]. qPCR was performed on an ABI StepOnePlus device (Applied Biosystems) in paired reactions using the Taqman gene expression assays with following ID numbers (all from Applied Biosystems): IL-6, Hs00985639_m1; CXCL8, Hs00174103_m1; CCL2, Hs00234140_m1; β-actin, Hs99999903_m1. qPCR reactions were performed in triplicate in 96-well plates using the following thermocycling conditions: 95°C for 10 min; 40 cycles, each for 15 s at 95°C and at 60°C for 1 min. The point at which the PCR product was first detected above a fixed threshold (cycle threshold, C_t_), was determined for each sample. Changes in the expression of target genes were calculated using the 2^-ΔΔCt^ method, where ΔΔC_t_ = (C_t_^target^-C_t_^β-actin^)_sample_-(C_t_^target^-C_t_^β-actin^)_control_, taking an untreated sample as a control.

Commercially available ELISA Ready-Set-Go! kits (eBioscience, San Diego, CA, USA) were used for measurements of IL-6, CXCL8, and CCL2 levels in the conditioned media.

### Statistical Analysis

**Fig 1:**
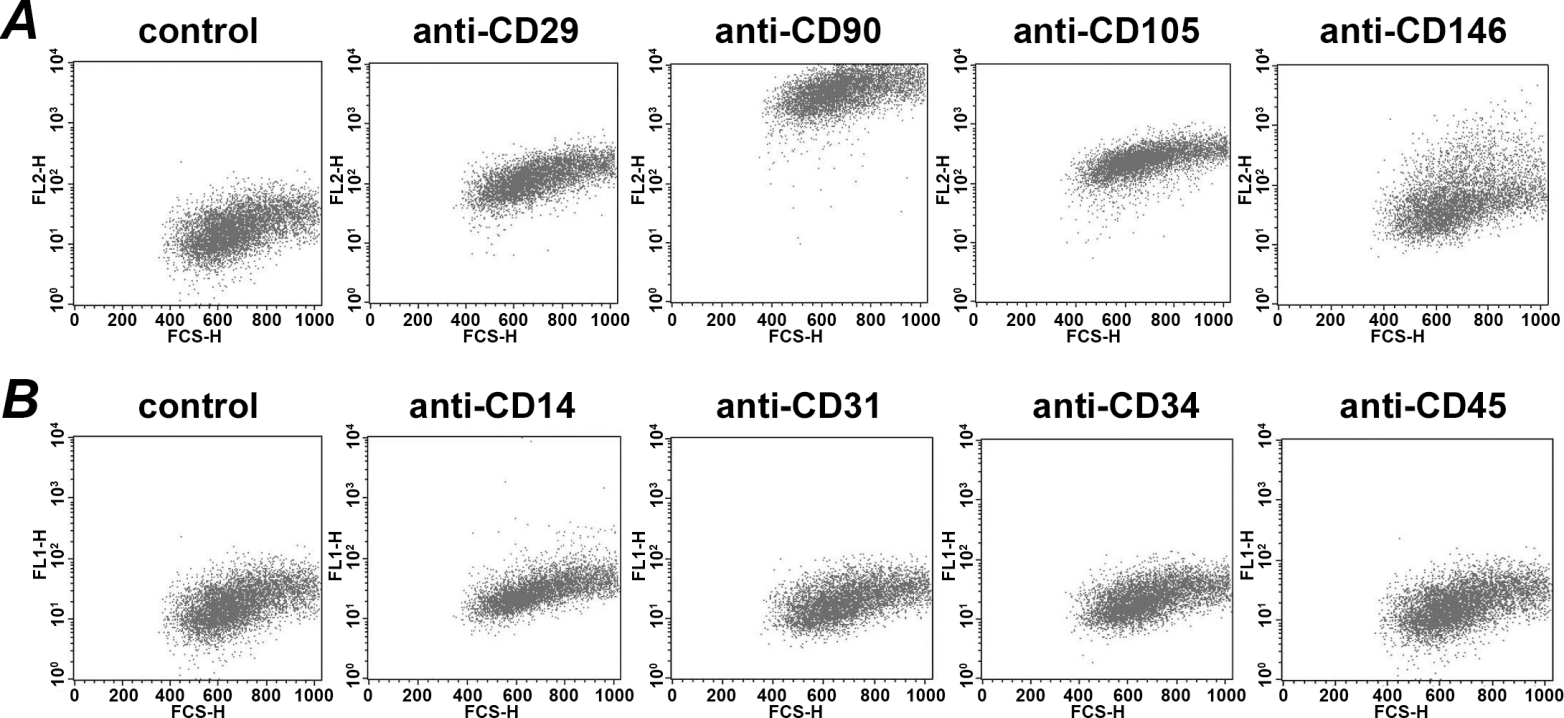
Representative Expression of surface markers on human periodontal ligament stem cells. Cells were stained with specific antibodies and the expression of different surface markers was analyzed by flow cytometry. A–staining with mesenchymal markers CD29, CD90, CD105, CD146 and corresponding isotype control antibody. B–staining with hematopoietic markers CD14, CD31, CD34, and CD45.

After confirming normal distribution by Kolmogorov-Smirnov test, the statistical differences between different groups were analyzed by one-way analysis of variance (ANOVA) for repeated measures followed by t-test. All statistical analyses were performed using statistical program SPSS 21.0 (SPSS, Chicago, IL, USA). Data are expressed as mean ± S.E.M. Differences were considered to be statistically significant at p < 0.05. All figures excluding Fig 1 represent a pooled data of 7 different donors.

## Results

### Expression of surface markers and differentiation ability of PDLSCs

The representative expression of specific markers on the hPDLSCs surface is shown in Fig 1. As can be seen, the cells were positively stained to mesenchymal stem cells markers and negatively stained to hematopoietic markers. The following proportions of positively stained cells with mesenchymal markers were observed: CD29, 89.5±2.4; CD90, 99.6±0.1; CD105, 97.3±0.8; CD146, 34.2±3.2 (n = 7 for all markers). The following proportions of positively stained cells with hematopoietic markers were observed: CD14, 1.7±0.5; CD31, 0.9±0.2; CD34, 1.0±0.2; CD45, 1.1±0.1 (n = 7 for all markers).

### The effect of soluble CD14 on the response of hPDLSCs *P*. *gingivalis* LPS, *E*. *coli* LPS, and Pam3CSK4

**Fig 2:**
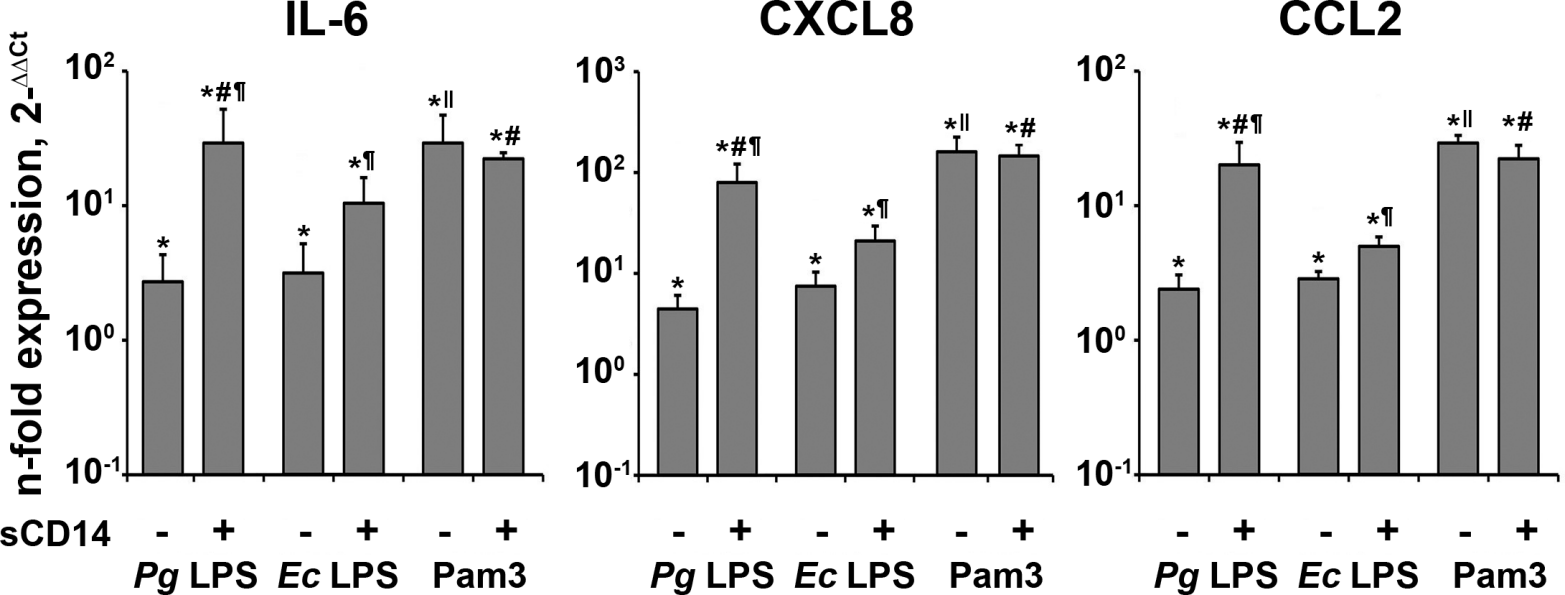
Gene-expression levels of IL-6, CXCL8, and CCL2 in hPDLSCs in response to stimulation with P. gingivalis LPS, E. coli LPS, and Pam3CSK4 in the presence or in the absence of sCD14. Human periodontal ligament stem cells were stimulated by *P*. *gingivalis* LPS (*Pg* LPS), *E*. *coli* LPS (*Ec* LPS), and Pam3CSK4 in concentration of 1 μg/ml for 24 h in the presence or in the absence of soluble CD14 (250 ng/ml) and the gene-expression levels of IL-6, CXCL8, and CCL2 were measured by qPCR. Y-axes represent the n-fold expression levels of target gene in relation to unstimulated cells (control = 1). Data are presented as mean ± S.E.M. of 7 independent experiments with cells isolated from 7 different donors. *—significantly higher than control (non-stimulated cells), p<0.05. #—significantly higher than *Ec* LPS with sCD14 group, p<0.05. ¶ - significantly higher than group without sCD14, p<0.05. ǁ - significantly higher than *Pg* LPS without sCD14 and *Ec* LPS without CD14 groups, p<0.05.

The effect sCD14 (250 ng/ml) on the gene expression levels of IL-6, CXCL8, and CCL2 in hPDLSCs on response to different stimuli is shown in Fig 2. As can be seen, every stimulus induced a significant increase in the expression of all genes in hPDLSCs. In the absence of sCD14, the response of hPDLSCs to both *P*. *gingivalis* LPS and *E*. *coli* LPS was lower than that to Pam3CSK4. In the presence of sCD14, the response of hPDLSCs to both types of LPS was significantly increased (p<0.05). Under these conditions, the response to *P*. *gingivalis* LPS was significantly higher than that to *E*. *coli* LPS. The response to TLR2 agonist Pam3CSK4 (1 μg/ml) was not dependent on the presence of sCD14. In the presence of sCD14, the response to Pam3CSK4 was similar to that of *P*. *gingivalis* LPS and significantly higher compared to *E*. *coli* LPS.

**Fig 3:**
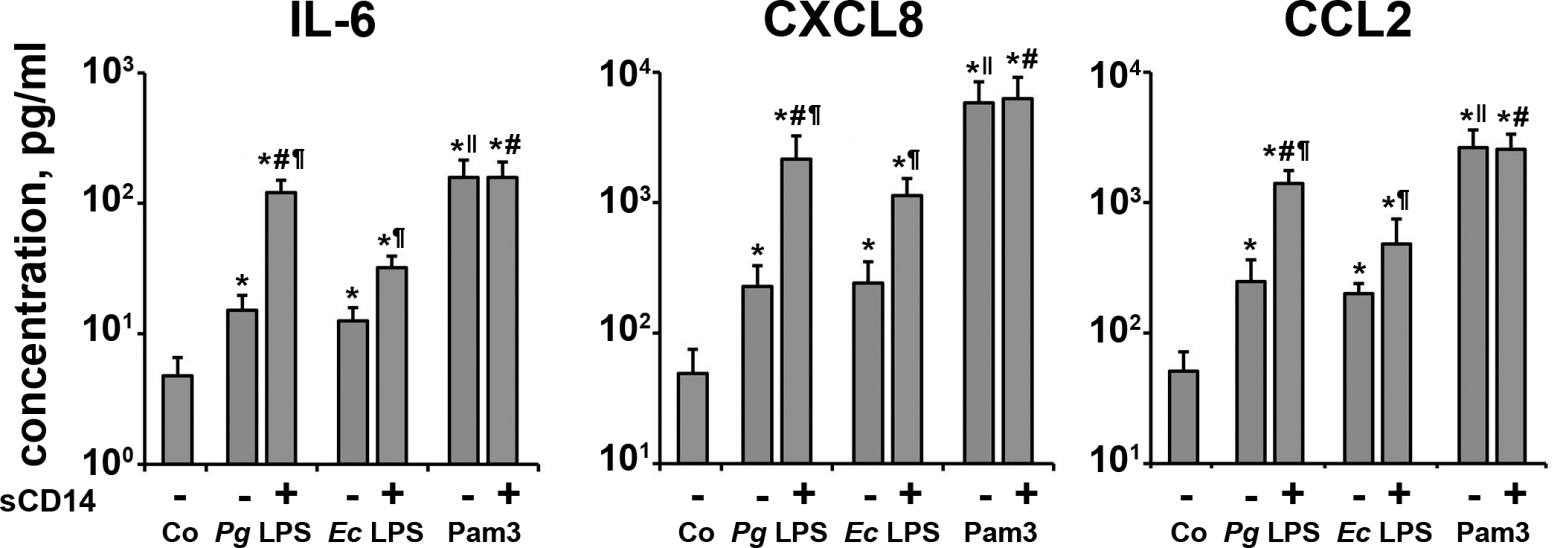
Production of IL-6, CXCL8, and CCL2 by hPDLSCs in response to stimulation with P. gingivalis LPS, E. coli LPS, and Pam3CSK4 in the presence or in the absence of sCD14. Human periodontal ligament stem cells were stimulated by *P*. *gingivalis* LPS (*Pg* LPS), *E*. *coli* LPS (*Ec* LPS), and Pam3CSK4 in concentration of 1 μg/ml for 24 h in presence or absence of soluble CD14 (250 ng/ml) and the concentration of IL-6, CXCL8, and CCL2 in conditioned media were measured by ELISA. Concentrations of IL-6, CXCL8, and CCL2 in conditioned media of unstimulated cells were taken as control (Co). Data are presented as mean ± S.E.M. of 7 independent experiments with cells isolated from 7 different donors. *—significantly higher than control (non-stimulated cells), p<0.05. #—significantly higher than *Ec* LPS with sCD14 group, p<0.05. ¶ - significantly higher than group without sCD14, p<0.05. ǁ - significantly higher than *Pg* LPS without sCD14 and *Ec* LPS without CD14 groups, p<0.05.

The content of IL-6, CXCL8, and CCL2 in the conditioned media upon stimulation with *P*. *gingivalis* LPS, *E*. *coli* LPS, and Pam3CSK4 (all 1 μg/ml) in the presence or in the absence of sCD14 (250 ng/ml) is shown in Fig 3. The measurements of protein levels were in agreement with the data on the gene expression. The content of all three proteins in the conditioned medium in response to stimulation with both types of LPS was enhanced by sCD14. In the absence of sCD14, the response to both *P*. *gingivalis* LPS and *E*. *coli* LPS was significantly lower than that to Pam3CSK4. In the presence of sCD14, the response to *P*. *gingivalis* LPS and Pam3CSK4 was significantly higher than that to *E*. *coli* LPS. No significant effect of sCD14 on the protein production by Pam3CSK4 was observed. In the presence of sCD14, no significant difference in the protein production between stimulation with *P*. *gingivalis* LPS and Pam3CSK4 was observed.

### Dependency of the response of hPDLSCs to *P*. *gingivalis* LPS on the concentration of sCD14

**Fig 4:**
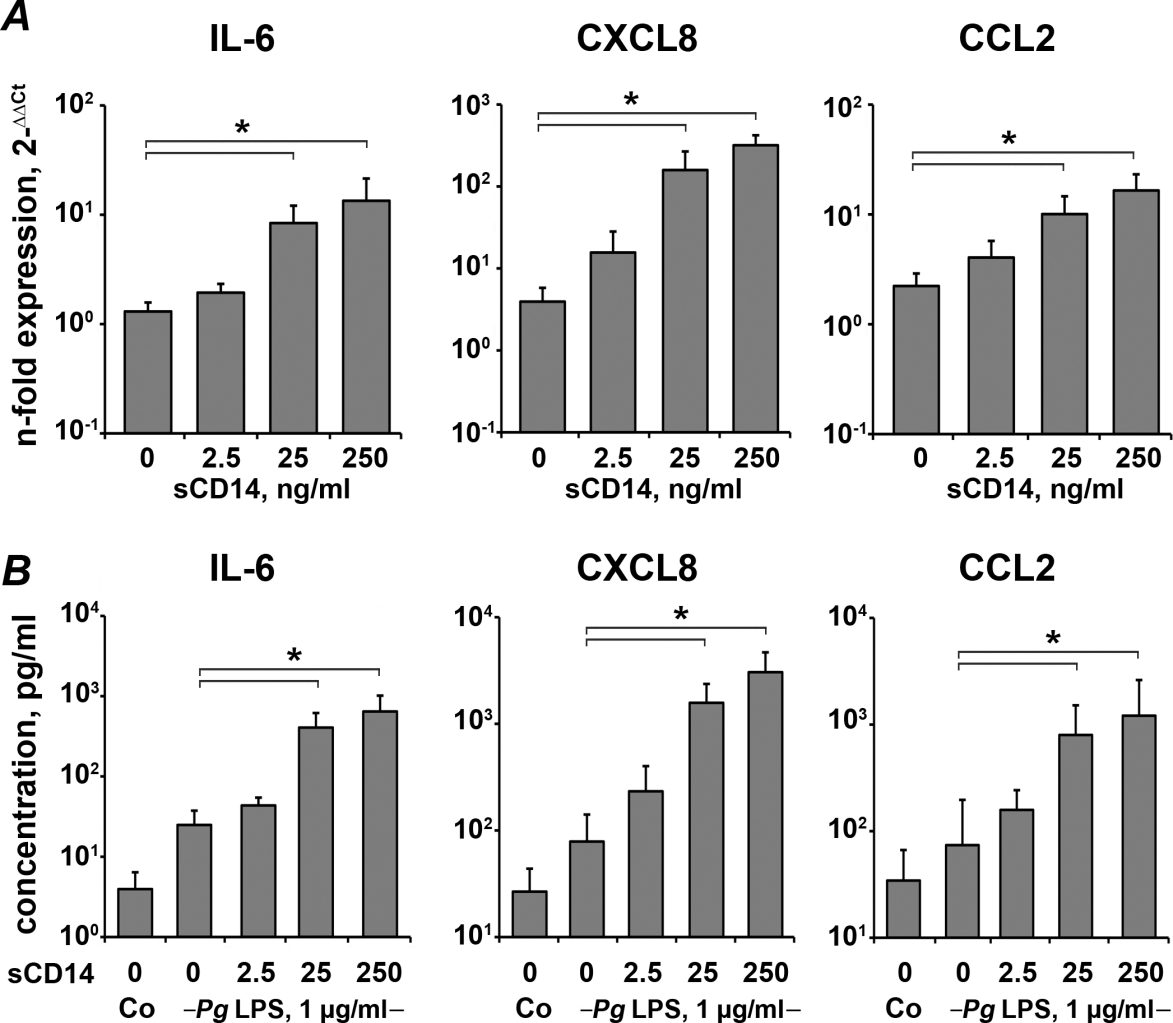
Response of hPDLSCs to stimulation with P. gingivalis LPS in the presence of different amounts of sCD14. Human periodontal ligament stem cells were stimulated by *P*. *gingivalis* LPS in concentration of 1 μg/ml for 24 h in the presence of different amounts of sCD14 ranging from 0 to 250 ng/ml. A–gene expression levels of IL-6, CXCL8, and CCL2 measured by qPCR. Y-axes represent the n-fold expression levels of target gene in relation to unstimulated cells (control = 1). B–the concentrations of IL-6, CXCL8, and CCL2 proteins in the conditioned media measured by ELISA. Data are presented as mean ± S.E.M. of 7 independent experiments with cells isolated from 7 different donors. *—significantly different between groups, p<0.05.

The dependency of the response of hPDLSCs to *P*. *gingivalis* LPS on the sCD14 in the range from 0 to 250 ng/ml is shown in Fig 4. As can be seen, no significant effect of sCD14 in the concentration of 2.5 ng/ml on the hPDLSc response to *P*. *gingivalis* LPS was observed. Higher concentration of sCD14 (25–250 ng/ml) induced a significant increase in the expression of IL-6, CXCL8, and CCL2 in response to *P*. *gingivalis* LPS (1 μg/ml). This was observed on both gene and protein levels. No significant difference between the effect of sCD14 in concentration of 25 ng/ml and 250 ng/ml on the response of hPDLSCs to *P*. *gingivalis* LPS was observed.

### Concentration-dependent response of hPDLSCs to *P*. *gingivalis* LPS in the presence of CD14

**Fig 5:**
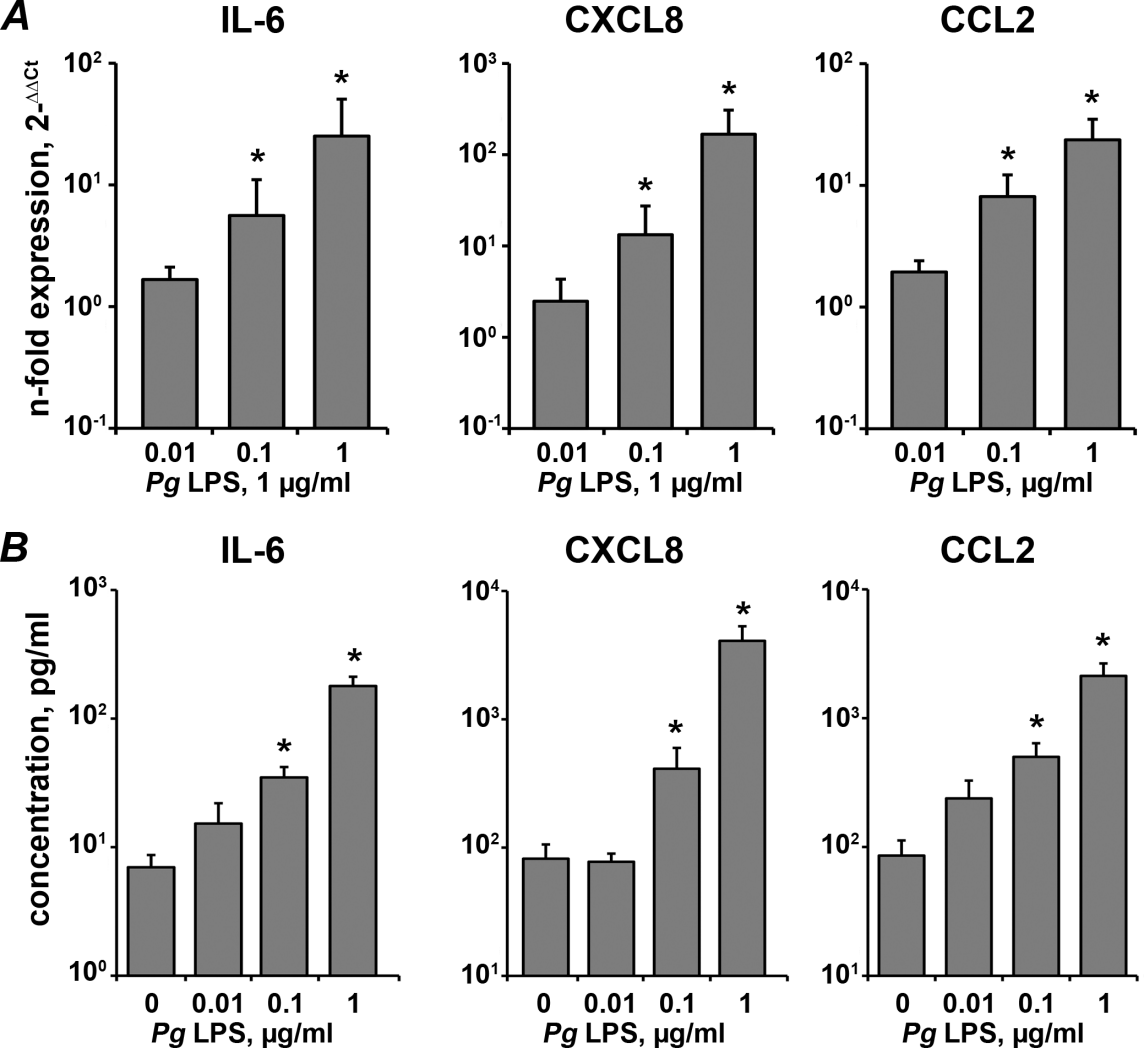
Response of hPDLSCs to stimualtion with different concentrations of P. gingivalis LPS in the presence of sCD14. Human periodontal ligament stem cells were stimulated by *P*. *gingivalis* LPS in concentrations ranging from 0.01 μg/ml to 1 μg/ml for 24 h in the presence of sCD14 in concentration of 250 ng/ml. A–gene expression levels of IL-6, CXCL8, and CCL2 measured by qPCR. Y-axes represent the n-fold expression levels of target gene in relation to unstimulated cells (control = 1). B–the concentrations of IL-6, CXCL8, and CCL2 proteins in the conditioned media measured by ELISA. Data are presented as mean ± S.E.M. of 7 independent experiments with cells isolated from 7 different donors. *—significantly higher than control (non-stimulated cells), p<0.05.

Fig 5 shows the production of IL-6, CXCL8, and CCL2 by hPDLSCs in response to stimulation with different concentrations of *P*. *gingivalis* LPS (0.01–1 μg/ml) measured in the presence of sCD14. As can be seen, significantly increased expression of all three proteins was observed already after stimulation with *P*. *gingivalis* LPS in concentration of 0.1 μg/ml. No significant difference in the expression of all proteins was observed after stimulation with *P*. *gingivalis* LPS at a concentration of 0.01 μg/ml.

## Discussion

In the present study, we investigated the effect of *P*. *gingivalis* LPS on the production of IL-6, CXCL8, and CCL2 by human periodontal ligament stem cells. The effect of bacterial LPS on the dental-derived MSCs is investigated in some previous studies but their results are rather controversial (for review, see [25]). Particularly, different effects of LPS on the differentiation potential and the production of pro-inflammatory factors are reported, and some studies do not observe any effect of LPS on these parameters [25]. We hypothesized that the effect of *P*. *gingivalis* LPS on the hPDLSC might be enhanced by soluble CD14 (sCD14). Our data suggest that in the presence of exogenous sCD14 *P*. *gingivalis* LPS induces a strong production of inflammatory mediators by hPDLSC, which might play an important role in the inflammatory response during periodontitis.

Membrane bound CD14 (mCD14) is a GPI-anchored protein on the cell surface [26]. Beside the membrane-bound form, there is also a soluble form of CD14 (sCD14) [21]. The major function of CD14 is to facilitate binding of LPS to the transmembrane TLR4 receptor [27]. Another function of CD14 is a control of TLR4 internalization and activation of MyD88 independent pathway [28]. In agreement with these criteria of MSCs [1], we did not observe the expression of CD14 on the surface of PDLSCs. The proportion of CD14^+^ cells in our suspension was about 1.7%, which was slightly higher compared to a recent study showing that a population of CD14 positive cells in PDLSCs suspension does not exceed 0.3% [29]. This differences might be explained by the observation that mesenchymal stem cells express some CD14 reactive epitopes and might bind some CD14 antibodies despite they do not express CD14 protein [30]. The absence of surface expression of mCD14 in hPDLSCs can explain the relatively low response of these cells to bacterial LPS. The response of hPDLSc to both types of LPS was significantly enhanced by soluble CD14. Soluble CD14 facilitate interaction of LPS with membrane TLR4 [31], whereas there is no evidence that sCD14 facilitate activation of TLR2. Therefore, we assume that a sCD14-dependent response to bacterial LPS could be mediated mainly by TLR4.

Although the response of both *P*. *gingivalis* LPS and *E*. *coli* LPS was markedly enhanced by sCD14, some differences in the response of hPDLSCs to these different LPS might be detected. In the absence of sCD14, both *P*. *gingivalis* LPS and *E*. *coli* LPS induced a production of IL-6, CXCL8, and CCL2, which was however, significantly lower than that to TLR2 agonist Pam3CSK4. Since hPDLSc do not express mCD14, it can be assumed that this activation occurs in a CD14-independent manner. The ability of *E*. *coli* LPS to activate the host response in CD14-independent manner was already shown in studies using blocking anti CD14 antibodies as well as on CD14-deficient animals [32,33]. In the present study, we used a standard commercially available *P*. *gingivalis* LPS preparation. Although some studies report that this preparation is free of lipid contamination [34], the presence of some lipid traces, which are potential activators of TLR2, is still assumed by the supplier. Therefore, it cannot be excluded that these contaminations contribute to hPDLSCs response to *P*. *gingivalis* LPS in the absence of sCD14. In the presence of sCD14, the response of hPDLSCs to *P*. *gingivalis* LPS was markedly higher than that of *E*. *coli* LPS. In other words, potentiating effects of sCD14 is higher for *P*. *gingivalis* LPS than for *E*. *coli* LPS. The reason for this observation is not entirely clear. First, structural differences between *P*. *gingivalis* LPS and *E*. *coli* LPS [13,35] might be responsible for the different effects of sCD14 on their interaction with TLR4. Second, it is possible that some contaminating lipids in *P*. *gingivalis* LPS preparation, which are potential activators of TLR2, might augment sCD14-mediated response to *P*. *gingivalis* LPS. This hypothesis is supported by the observation that interaction ofTLR2 and TLR4 might play an important role in LPS-induced TLR4-mediated response [36].

In the presence of sCD14, a significant increase in the production of IL6, CXCL8, and CCL2 is observed already after stimulation with *P*. *gingivalis* LPS at the concentration of 0.1 μg/ml. This concentration range is lower than working concentration of *P*. *gingivalis* LPS observed in other studies on stem cells of dental origin, in which exogenous sCD14 was not added. Activation of NF-kappaB in human PDLSCs and bone marrow MSC is induced by *P*. *gingivalis* LPS at a concentration of 10 μg/ml [37]. In human dental pulp stem cells *P*. *gingivalis* LPS at a concentration of 1 μg/ml induced only modest changes in the phosphorylation of IkBa [38]. A study on dental follicle progenitor cells reports that *P*. *gingivalis* LPS in concentrations up to 50 μg/ml does not influence the production of IL-6 by these cells [39]. LPS isolated from other species also influences dental stem cell only at high concentrations in the absence of exogenous sCD14. Particularly, an inhibition of osteogenic differentiation of hPDLSC by *E*. *coli* LPS was observed at concentration of 10 μg/ml [40]. Thus, the presence of sCD14 increases the affinity of dental stem cells to bacterial LPS and they can sense lower LPS concentrations.

The dependency of the hPDLSC response to bacterial LPS on sCD14 suggests that the local levels of this protein might play an important role in the progression of periodontal disease. Clinical studies show that the local and systemic CD14 levels are substantially affected by periodontal disease. Particularly, periodontitis patients with higher levels of sCD14 in GCF have fewer deep pockets [41]. The same study reports a negative correlation between sCD14 level in GCF and periodontal pocket depth [41]. Another study shows that the levels of sCD14 in whole saliva is higher in periodontitis patients than in healthy controls and that salivary CD14 level exhibit a significant positive correlation with clinical measurements of periodontitis [42]. Finally, serum sCD14 levels are higher in patients with periodontitis than in healthy subjects [43,44].

The physiological role of the cytokine production by hPDLSCs in response to bacterial LPS still remains to be elucidated. Two different aspects might be especially taken into consideration. On the one hand, the production of IL-6, CXCL8, and CCL2 might promote inflammatory response in periodontitis. We focused on the measurements on the expression of IL-6, CXCL8, and CCL2, which are thought to play an important role in the progression of periodontal disease. IL-6 is a pro-inflammatory cytokine, which plays a key role in acute inflammation phase and promotes bone resorption [45]. CXCL8 (also called interleukin 8) and CCL2 (also called monocyte chemoattractant protein 1) are chemoattractants, which induce migration of neutrophils and monocytes, respectively, to the inflammation site and promote the development of acute inflammation [46,47]. On the other hand, recent studies suggest that IL-6 and CCL2 might be involved in the MSC-mediated immunosuppression. Particularly, IL-6 might be involved in T-cell suppression [48], whereas CCL2 might mediate MSC-induced T-cell apoptosis [49]. Some previous studies show that immunomodulatory properties of MSCs are influenced by TLRs activation [50], which might play a role in the progression of periodontitis. This assumption is strengthened by studies showing that hPDLSCs isolated from periodontitis patient possess impaired immunomodulatory abilities [51,52].

The response of periodontal ligament stem cells to the *P*. *gingivalis* LPS in the presence of soluble CD14 is observed already at the concentration range of ng/ml and therefore might be physiologically relevant. The levels of sCD14 used in our study seem to be also physiologically relevant. A previous study reports that sCD14 concentration in GCF ranges from 0.16 to 51.74 μg/ml [41]. Another study reports that the levels of sCD14 in blood serum are in the range of few μg per milliliter [43]. Changes in the levels of sCD14 might be an important mechanism influencing the response to periodontal pathogen, but its exact role need to be further investigated. Moreover, the presence of sCD14 might also influence the regenerative potential of resident MSC-like cells of dental tissues, particularly their proliferation and differentiation ability. The effect of sCD14 on these parameters must be investigated in future studies.

Summarizing, our data show that the response of human periodontal ligament stem cells (hPDLSCs) to bacterial lipopolysaccharide (LPS) is significantly enhanced by soluble CD14. CD14 is an important factor involved in the activation of TLR4 by bacterial LPS and hPDLSCs usually are not expressing this protein on their surface. In the presence of physiologically relevant levels of soluble CD14, hPDLSCs exhibit a significant response even to rather low LPS concentrations. Changing in the extracellular levels of soluble CD14 might influence the responsiveness of hPDLSC to different pathogens and might have a potential effect on their immunomodulatory properties, which might play an important role in the pathogenesis of periodontitis.
